# Alcohol calibration of tests measuring skills related to car driving

**DOI:** 10.1007/s00213-013-3408-y

**Published:** 2014-01-10

**Authors:** Stefan Jongen, Eric Vuurman, Jan Ramaekers, Annemiek Vermeeren

**Affiliations:** Experimental Psychopharmacology Unit, Faculty of Psychology and Neuroscience, Maastricht University, Maastricht, The Netherlands

**Keywords:** Driving, Alcohol, Attention, Psychomotor performance, Cognition, Psychometric tests

## Abstract

**Rationale:**

Medication and illicit drugs can have detrimental side effects which impair driving performance. A drug’s impairing potential should be determined by well-validated, reliable, and sensitive tests and ideally be calibrated by benchmark drugs and doses. To date, no consensus has been reached on the issue of which psychometric tests are best suited for initial screening of a drug’s driving impairment potential.

**Objective:**

The aim of this alcohol calibration study is to determine which performance tests are useful to measure drug-induced impairment. The effects of alcohol are used to compare the psychometric quality between tests and as benchmark to quantify performance changes in each test associated with potentially impairing drug effects.

**Methods:**

Twenty-four healthy volunteers participated in a double-blind, four-way crossover study. Treatments were placebo and three different doses of alcohol leading to blood alcohol concentrations (BACs) of 0.2, 0.5, and 0.8 g/L.

**Results:**

Main effects of alcohol were found in most tests. Compared with placebo, performance in the Divided Attention Test (DAT) was significantly impaired after all alcohol doses and performance in the Psychomotor Vigilance Test (PVT) and the Balance Test was impaired with a BAC of 0.5 and 0.8 g/L. The largest effect sizes were found on postural balance with eyes open and mean reaction time in the divided attention and the psychomotor vigilance test.

**Conclusions:**

The preferable tests for initial screening are the DAT and the PVT, as these tests were most sensitive to the impairing effects of alcohol and being considerably valid in assessing potential driving impairment.

## Introduction

Many people use drugs other than alcohol (i.e., medicinal and illicit drugs) which can impair performance (e.g., Vingilis and Macdonald 2002; Walsh et al. 2004). In Europe, a study, which was a part of the *DRiving Under the Influence of Drugs, alcohol and medicines (DRUID)* project, reported an estimated prevalence of alcohol use of 3.48 %, illicit drug use of 1.90 %, impairing medicinal drug use of 1.36 %, and drug-drug combination of 0.39 % in the general population of drivers (Houwing et al. 2011). A roadside survey in the USA indicated that illicit drugs are used by 5.8 %, medicinal drugs by 4.8 %, and a drug-drug combination by 0.5 % of weekend drivers (Lacey et al. 2009). Because of the large number of people involved and the severity of the consequences, one of the most important risks of medicinal and illicit drug use is that of impaired driving performance and traffic accidents (e.g., O’Hanlon et al. 1982; O’Hanlon 1984; Seppala et al. 1979). Therefore, a standardized scientific evaluation of potential drug-induced performance impairment is needed to provide more meaningful warnings to users and prescribers regarding the impacts of drugs on driving (Kay and Logan 2011). In addition, there is an increasing demand from regulatory authorities to provide more information on the risks of drug-induced impairment of driving. This concerns not only newly developed medicinal drugs as part of the dossier for registration but also marketed and illicit drugs to determine thresholds for drug concentrations in blood associated with driving impairment (e.g., Verstraete et al. 2011).

Methodological guidelines for experimental studies assessing the effects of drugs on driving indicated that relatively simple tests may be used as a first step in screening a drug’s impairment potential and that more sophisticated procedures (e.g., driving simulators, on-the-road testing) should be included in a later stage (ICADTS 1999; Kay and Logan 2011; Vermeeren et al. 1994; Walsh et al. 2008). Among the more sophisticated tests, the standardized highway driving test developed by O’Hanlon and colleagues is generally regarded as the gold standard to measure drug-induced driving impairment (O’Hanlon 1984; Verster and Roth 2011). However, no consensus has been reached on the specific tests to be used for initial screening. Such tests have to be widely available, easy to implement, and relatively cost effective. In order to provide relevant information, they should be valid with respect to measuring driving performance supported by theoretical models of driving (e.g., Michon 1989), have reasonable test-retest reliability, and be sufficiently sensitive to detect drug-induced impairment (Kay and Logan 2011; Walsh et al. 2008). To ensure comparability of results from various research settings, procedures should be standardized and results calibrated by benchmark drugs and doses.

The most well-known and widely used benchmark drug for assessing drug-induced driving impairment is alcohol (e.g., González-Wilhelm 2007; Louwerens et al. 1987). The increased risk of traffic accidents is well established for various legal limits of blood alcohol concentrations (BACs). Although some epidemiological studies found that the risk of being involved in a fatal crash for drivers at BACs even as low as 0.2–0.4 g/L is increased (Zador et al. 2000), most studies found that risks increase exponentially with BACs of 0.5 g/L and higher (e.g., Borkenstein 1964; Krüger 1993; Schnabel et al. 2010). In line with this, the legal BAC limit for driving a car is set at a BAC of 0.5 g/L in most countries, but differences are applicable. For example, the legal limit is 0.2 and 0.8 g/L in Sweden and the UK, respectively. Although legal limits are societal issues, it is generally agreed that the impairment produced by BACs of 0.5 g/L and higher is clinically relevant. Therefore, a BAC of 0.5 g/L can be used to calibrate performance changes in tests measuring driving-related skills. Drug effects comparable to those of alcohol with BACs between 0.5 and 0.8 g/L are generally classified as moderately severe, whereas drug effects comparable with BACs over 0.8 g/L are classified as severe.

Review of the literature shows that a number of psychometric tests are preferably used to assess possible driving impairment, but the choice of tests differs depending on the area of research or practice. Experimental studies assessing effects of drugs and alcohol on driving and driving-related skills in healthy volunteers concluded that tracking performance and divided attention, as measured by the Critical Tracking Test (CTT) and the Divided Attention Test (DAT), are among the most sensitive tests (e.g., Moskowitz 1973; Ramaekers et al. 2003; Robbe and O’Hanlon 1995; Verster and Roth 2011). Clinical trials assessing pharmacokinetics and pharmacodynamics of new medicinal drugs usually include the Digit Symbol Substitution Test (DSST) to determine side effects of various doses on daytime functioning (e.g., Greenblatt et al. 2006; Roth et al. 2008). In sleep research, the Psychomotor Vigilance Test (PVT) is nowadays considered as gold standard for assessing driver drowsiness resulting from disturbed or insufficient sleep (e.g., Doran et al. 2001; Jewett et al. 1999). In the field of aging and dementia, tests measuring processing speed and cognitive flexibility, such as the Trail Making Test (TMT) or the Concept Shifting Test (CST), and Digit Span Test (DST) to measure memory span, are often considered to be good predictors of on-road-driving performance (e.g., Clark et al. 2011; Silva et al. 2009). In the field of neuropsychology, a frequent used test is the Attention Network Test (ANT) (e.g., Weaver et al. 2009), because it measures the efficiency of multiple attention networks in a relatively short time. Furthermore, balance tests as part of a Standardized Field Sobriety Test (SFST) are commonly used at the roadside by trained policemen (i.e., Drug Recognition Experts) in the USA and Australia to detect drug-induced driving impairment (Stuster and Burns 1998).

The list of tests measuring driving-related and drug-induced impairment is exhaustive. Therefore, not all available tests could be included; however, we aimed to compare a number of widely used tests representative for different fields of research related to driving performance and traffic safety. The main objective of this study was to compare the relative sensitivity of these tests for the dose-dependent effects of alcohol. Since the effects of alcohol are relatively nonspecific it is expected to affect performance in most tests, in particular with a BAC of 0.8 g/L. More sensitive tests and parameters are assumed to show larger effect sizes and significant effects at lower BACs. A secondary objective was to establish the mean performance changes in each test associated with three increasing doses of alcohol resulting in BACs of 0.2, 0.5, and 0.8 g/L for future reference and interpretation of the clinical relevance of drug effects, which could provide a comparison of a full range of driving-related skills.

## Methods

### Participants

Twenty-five healthy male and female volunteers (ages 18–30 years) were recruited through poster advertisements at Maastricht University. Initial screening was based on a medical history questionnaire examined by a medical supervisor. Accepted participants underwent a physical examination, which included standard blood hematology and chemistry, urinalysis, pregnancy tests, and tests for drugs of abuse (amphetamines, benzodiazepines, cannabis, cocaine, 3,4-methylenedioxymethamphetamine, and opiates). For participation, the following inclusion criteria had to be met: social drinking, defined as drinking at least three but no more than 21 glasses of alcohol, per week; and a Body Mass Index (BMI) between 19 and 29 kg/m^2^. Exclusion criteria included pregnancy or lactation; any history of psychiatric or medical illness; history or current drug or alcohol abuse; current use of psychoactive medication; inability to stay abstinent during the study; excessive caffeine use, defined as drinking six or more cups of coffee per day; and habitual smoking, defined as smoking more than seven cigarettes a week.

One participant dropped out after the familiarization session for reasons unrelated to treatment. A total of 24 participants (12 men, 12 women) completed the study. The mean (±SD) age was 22.7 (±2.3) years and their mean (±SD) BMI was 22.5 (±2.0) kg/m^2^. The study was conducted in accordance with the code of ethics on human experimentation established by the Declaration of Helsinki (1964) and amended in Seoul (2008). All participants were informed of the study’s goal, procedures, and potential hazards in writing, and they indicated their informed consent in writing. The Medical Ethics Committees of Maastricht University approved the study. Participants received a financial compensation for their participation in the study.

### Design and alcohol administration

The study was conducted according to a double-blind, placebo-controlled, four-way crossover design. Treatments were alcohol doses to reach BACs of 0.0 (i.e., placebo), 0.2, 0.5, and 0.8 g/L. Volunteers participated in two treatment days during which two doses of alcohol were administered each day. The first dose was to achieve a low BAC (i.e., 0.0 or 0.2 g/L) and the second dose was to achieve a high BAC (i.e., 0.5 or 0.8 g/L). The order of test days was balanced over participants. The washout period was at least 1 week.

**Fig. 1:**
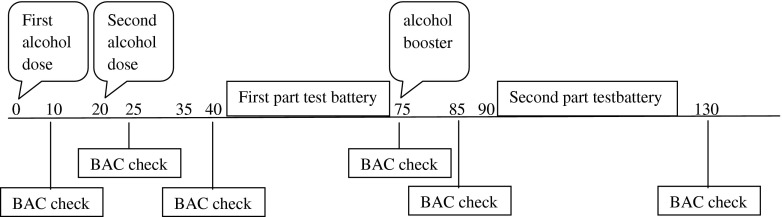
Timeline in minutes for alcohol administration, test performance, and obtaining BAC samples

The placebo dose consisted of a glass of orange juice with a small amount (3 mL) of alcohol floating on the surface of the beverage. This is an effective procedure which indicates that the beverage contained alcohol (e.g., Fillmore and Vogel-Sprott 1998). In the other conditions, participants were treated with several alcohol (97 %) challenges mixed with orange juice. Alcohol was administered orally. The alcohol dosing regimen was developed to reach BACs of 0.0, 0.2, 0.5, and 0.8 g/L. To verify this, breath samples were obtained with an alcohol breathalyzer (Dräger Alcotest 6510). The required quantity of alcohol to reach the aforementioned BACs depended on gender and weight of the individual. It was calculated by an improved version (Watson 1981) of the" “Widmark formula” (Widmark 1932). Within the improved formula, the amount of alcohol is related to the individual’s total body water content. With consideration of the specific weight of alcohol and the BAC, the necessary quantity is calculated and divided over two glasses. Participants were allowed 5 min per glass to consume the drinks. A breath sample was obtained 10 min after the intake of each glass of alcohol. If necessary, an additional dose of alcohol was given to the participant. A breath sample was again obtained before testing started. Halfway the test session, the BAC was measured again, and an additional alcohol dose was administered if BAC had decreased below 0.1 g/L of the required BAC. Figure 1 indicates a timeline of a testing day.

### Procedure

Participants were individually trained to perform the behavioral tests prior to the first test day. The behavioral tests were conducted twice on two separate days. Tests were always administered according to the same order: i.e., Concept Shifting Test, Critical Tracking Test, Divided Attention Test, Psychomotor Vigilance Test, Digit Symbol Substitution Test, Digit Span Test, Attention Network Test, and Postural Balance Test.

During participation in the study, alcohol intake was not allowed from 24 h prior to each dosing until discharge. On treatment days, caffeine intake and smoking were not allowed until discharge. Participants agreed not to use any drugs of abuse or oral medication (except oral contraceptives and aspirin) during the study.

On treatment days, participants fasted for 4 h before arrival at the lab. Participants yielded urine and breath samples prior to each test session to confirm their compliance with prohibitions against prior use of drugs and to verify a BAC of 0.0 g/L at the beginning of each session. Urine samples of women were examined to exclude pregnancy. A testing day ended at 2100 hours at which time participants were driven home.

### Assessment

#### Critical Tracking Test

The Critical Tracking Test (CTT) measures the ability to control an unstable error signal in a first-order compensatory tracking task (Jex et al. 1966). This test is designed to measure psychomotor coordination. Tracking skills are especially important at the operational/control level of driving behavior (e.g., keeping the car in a steady position within the lane). Participants are instructed to keep an unstable bar in the middle of a horizontal plane by counteracting or reverse its movements with the aid of a joystick. The frequency of cursor deviations at which the participant loses control is the critical frequency or lambda (*λ*_c_, in rad/s). The final score is determined from the average of all but the lowest and highest scores in five trails. Total duration of the test is approximately 3 min.

#### Divided Attention Test

The Divided Attention Test (DAT) measures the ability to perform two tasks simultaneously (Moskowitz 1973). In the primary task, the participants perform the same tracking task described above, set at a constant level of difficulty. In the other task, the participant monitors 24 peripheral displays in which single digits change asynchronously at 5-s intervals. Participants are instructed to remove their foot from a pedal as rapidly as possible whenever the digit “2” appears. This signal occurs twice at every location, in random order, at intervals of 5–25 s. Tracking error (DAT-ER, in mm) and average reaction time to targets (DAT-RT, in ms) are the respective performance measures. Duration of the task is 12 min.

#### Psychomotor Vigilance Test

The Psychomotor Vigilance Test (PVT) is based on a simple visual reaction time test (Dinges and Powell 1985). The test measures ability to sustain attention over a period of 10 min. Participants are required to respond to a visual stimulus presented at a variable interval (2,000 to 10,000 ms) by pressing a button with the dominant hand. The visual stimulus is a counter turning on and incrementing from 0 to 60 s at 1-ms intervals. If a response has not been made in 60 s, the clock resets and the counter restarts. The reciprocal transform of the reaction time (1/RT) was calculated because it emphasizes slowing in the optimum and intermediate response domain and substantially decreases the contribution of long lapses. For calculation of mean 1/RT, each RT (ms) was divided by 1,000 and then reciprocally transformed. This measure has shown to indicate the largest effect sizes when taking mean reaction times into account (Basner and Dinges 2011).

#### Attention Network Test

The Attention Network Test (ANT) provides measures of three functions of attention within a single task: alertness, orienting, and executive control (Fan et al. 2002). Each trial begins with the presentation of a fixation cross in the middle of the computer screen. Participants are instructed to keep their eyes fixated on the cross throughout the test. Then, at some variable interval (ranging from 400 to 1,600 ms), a cue is presented for 100 ms. Four hundred milliseconds after the offset of the cue, a target display appears and remains until response (i.e., a key-press indicating the direction of the target arrow), or for 1,700 ms if no response is made. The interstimulus interval is 3,500 ms. There are four cue conditions and three target conditions. Targets (neutral, congruent, or incongruent) can appear either above or below the fixation cross. Dependent variables are total reaction time (RT) and differences between RTs reflecting efficiency of alerting (RT no cue − RT double cue), orienting (RT center cue − RT spatial cue), and executive networks (RT incongruent flankers − RT congruent flankers). Duration of the test is approximately 25 min.

#### Digit Symbol Substitution Test

The Digit Symbol Substitution Test (DSST) measures many different psychomotor and cognitive functions at the same time (Riedel et al. 2006). A computerized version of the original paper-and-pencil test (McLeod et al. 1982) taken from the Wechsler Adult Intelligence Scale is used (e.g., Leufkens et al. 2009). The participant is required to match each digit with a symbol from the encoding list as rapidly as possible by clicking the corresponding response button, using the mouse. The number of digits encoded correctly within 3 min is the performance measure.

#### Concept Shifting Test

The computerized Concept Shifting Test (CST) is used to measure processing speed and cognitive flexibility (Van der Elst et al. 2006). It consists of three subtasks (A, B, and C). On each display, 16 small circles are grouped into one larger circle. In the smaller circles, the test items appear in a fixed random order. Participants are asked to cross out numbers (1–16) in the right order as quickly and accurately as possible, using a touch screen. In part B, the circles contain letters (A–P) that have to be crossed out in alphabetical order. In part C, both numbers and letters are displayed, and the participant is requested to alternate between numbers and letters. The time needed to complete each part is scored in seconds (CST-A, CST-B, and CST-C, respectively). An interference score (CST_i_) was obtained by the following formula: (CST-C − ½ × (CST-A + CST-B)) / (½ × (CST-A + CST-B)) × 100. Total duration of the test is approximately 3 min.

#### Digit Span Test

The Digit Span Test (DST) measures memory span. It consists of two parts: one for forward digit span and one for backward digit span. In the forward part of the DST (Wechsler 1997), the experimenter reads various series of digits, 1–9, to the participant. The series are increased in length by one digit from trial to trial. Two sequences are presented for each span size. The instruction is to remember the digits and recall them immediately in correct serial order. Testing stops when the participant makes a mistake in both trials of the same span length. In the backward part of the DST, sequences of numbers are read out to participants who are asked to repeat them, in reverse order. The number of sequences correctly recalled forward and backward is recorded (DST-FW, DST-BW). Total duration of the test is approximately 2 min.

#### Postural Balance Test

The Postural Balance Test (PBT) measures the participant’s ability to maintain balance while standing upright on both feet. Balance is measured using the AMTI AccuSway System for Balance and Postural Sway Measurement (Advanced Mechanical Technology, Inc., Watertown, MA, USA) force platform (Mets et al. 2010, 2011). Postural sway is assessed by measuring the length of the path of the center of pressure (COP) and the area of the 95 % confidence ellipse enclosing the COP (A95). The test is conducted in two trials of both 60 s: one trial with the participants’ eyes open and one trial with eyes closed, both with feet apart at the hip’s width.

#### Subjective measures

Participants had to describe their subjective feeling in three dimensions (i.e., alertness, contentedness, and calmness) by using 16 Visual Analogue Scales (VAS) (Bond and Lader 1974). An additional scale was added on which participants could indicate their perceived degrees of intoxication. All scales were administered twice: once before testing started in a treatment condition and once afterward. After a testing day, participants were asked to estimate their level of intoxication (i.e., a BAC of 0.0, 0.2, 0.5, or 0.8 g/L) by a fixed choice question (i.e., “which BAC do you think we aimed to reach?”) for both test sessions.

### Statistical analyses

Sample size calculation was based on detecting a minimally relevant difference with an effect size of 0.25 between placebo and the 0.5 g/L BAC condition. Given a test-retest reliability of tracking error and reaction time at the Divided Attention Test of at least *r* = 0.75 (Ramaekers et al. 2011a), a group of 24 participants should permit detection of a mean change in tracking error and reaction time, with a power of at least 90 % and an *α* of 0.05.

First, correlations between the dependent variables from the eight tests in the placebo condition were calculated to determine differences or similarities in cognitive processes measured by each parameter.

Second, alcohol effects on each parameter were analyzed using general linear model repeated measures with alcohol (4 levels) as within-subjects factor. Three separate alcohol-placebo contrasts were conducted when an overall effect of alcohol was found. Bonferroni adjustment for the number of tests has been applied to correct for multiple comparisons (*α* = 0.05 / 3 = 0.0167).

Third, difference scores from placebo were calculated for the three active alcohol conditions to determine mean changes and 95 % confidence intervals for each parameter and alcohol dose.

Fourth, change scores for each of the dependent variables were transformed to *z*-scores, which were calculated across the pooled changes in the three active alcohol conditions. This allows for easy comparison across each of the various performance tests (Dry et al. 2012).

Finally, effect sizes were calculated to determine the magnitude of the effects of alcohol, using the effect size (ES) statistic (i.e., *t*_c_[2(1 − *r*) / *n*]^1/2^) for repeated measures of Dunlap et al. (1996). ESs between 0 and 0.2 are considered small, between 0.2 and 0.7 are considered moderate, and 0.7 or higher are considered large (Falleti et al. 2003). All statistical analyses were done by using the Statistical Package for the Social Sciences for Windows (version 19; SPSS Inc, Chicago, IL, USA).

## Results

### Missing data

Due to technical problems, no data were collected during the DAT on two occasions and during the CST on a single occasion. Only participants with complete data sets were entered into the analysis of the respective performance parameter.

### Blood alcohol concentrations

**Fig. 2:**
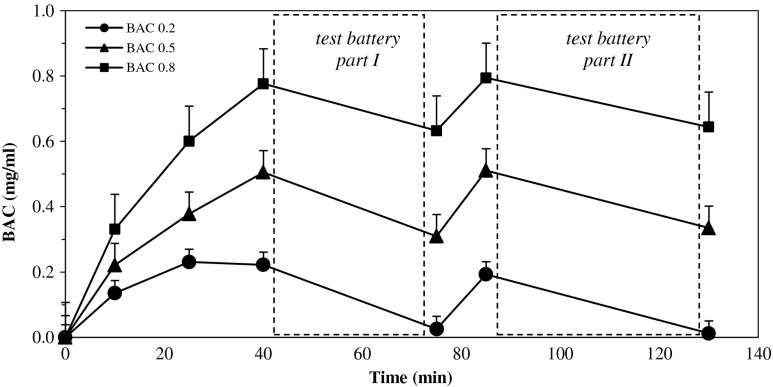
Mean blood alcohol concentrations (*BAC*) at each interval when breath samples were obtained. *Capped vertical lines* show standard errors of the mean

Figure 2 shows participants’ mean BAC at each interval when breath samples were obtained. Participants’ BAC generally peaked in the ranges of 0.16–0.27 g/L (mean ± SD, 0.22 ± 0.03 g/L), 0.45–0.56 g/L (0.51 ± 0.04 g/L), and 0.70–1.03 g/L (0.78 ± 0.07 g/L) within 40 min after drinking in the conditions to reach a BAC of 0.2, 0.5, and 0.8 g/L, respectively.

Mean BAC declined to 0.03, 0.31, and 0.63 g/L, respectively, over the course of the next 45 min. Booster doses were then administered to participants in whom the BAC reached below 0.1 g/L of the appropriate BAC. Mean BACs of 0.19 ± 0.04, 0.51 ± 0.04, and 0.79 ± 0.06 g/L were achieved 10 min later, and participants finished testing 45 min later with 0.01 ± 0.03, 0.34 ± 0.05, and 0.64 ± 0.05 g/L, respectively.

#### Correlations of performance measures

Correlations between the dependent variables from the eight tests measured in the placebo conditions showed that most correlations were low and not significant (−0.4 ≤ *r* ≤ 0.4). Absolute values of eight correlations were moderate (i.e., varying between 0.41 and 0.57). Performance in the PVT and DSST each correlated with performance in three other tests. Both correlated with overall reaction time in the ANT (*r* = 0.45 and *r* = −0.46, respectively). In addition, performance in the PVT correlated with tracking performance in the DAT (*r* = 0.49) and in the CTT (*r* = 0.48). Performance in the DSST, on the other hand, correlated additionally with reaction time in the DAT (i.e., target detection, *r* = 0.52) and with DST-forward (*r* = 0.46). Finally, tracking performance in the CTT correlated with tracking error in the DAT (*r =* −0.41), and the highest correlation was found between DST-forward and DST-backward (*r* = 0.57).

#### Alcohol effects

Table 1 presents a summary of the means and standard errors of the means (SE) of all performance scores and the results of the analyses of variance.

**Table 1:** Mean (SE), overall treatment effects, and contrast analyses of performance tests

Test	Mean (SE)				Overall treatment effect		Simple contrast analysis		
							PLA versus 0.2 g/L	PLA versus 0.5 g/L	PLA versus 0.8 g/L
	PLA	0.2 g/L	0.5 g/L	0.8 g/L	*F*	*p*	*p*	*p*	*p*
Critical Tracking Test									
Lambda (rad/s)	3.77 (0.12)	3.59 (0.14)	3.69 (0.13)	3.46 (0.13)	5.40	<0.01	<0.05	0.24	<0.001
Divided Attention Test									
Average error (mm)	15.2 (0.7)	16.0 (0.7)	15.0 (0.7)	17.0 (0.8)	2.70	0.07	0.18	0.73	<0.05
RT (ms)	1,873 (64.0)	1,996 (79.1)	1,988 (62.1)	2,082 (64.1)	5.71	<0.01	<0.05	<0.01	0.001
Psychomotor Vigilance Test									
1/RT (ms)	4.05 (0.1)	3.96 (0.1)	3.91 (0.1)	3.75 (0.1)	5.74	<0.01	0.14	<0.01	0.001
RT (ms)	261 (6.6)	268 (9.0)	275 (9.3)	286 (7.9)	6.49	<0.01	0.23	0.01	<0.001
Digit Symbol Substitution Test									
Correct responses	99.2 (3.2)	100.8 (2.7)	97.8 (3.0)	93.3 (2.2)	11.12	<0.001	0.57	0.49	<0.01
Attention Network Test									
Overall RT (ms)	468 (12.5)	470 (11.1)	473 (13.3)	498 (12.1)	9.93	<0.001	0.74	0.45	0.001
Alerting effect (ms)	63.8 (8.2)	53.6 (5.6)	67.0 (8.5)	75.7 (8.0)	2.25	0.11	0.22	0.72	0.27
Orienting effect (ms)	32.1 (4.8)	32.4 (3.8)	27.1 (4.4)	31.9 (5.1)	0.45	0.72	0.95	0.35	0.98
Conflict effect (ms)	77.8 (8.4)	80.2 (8.1)	86.3 (8.6)	98.9 (8.5)	2.24	0.11	0.64	0.42	0.07
Concept Shifting Test									
RT CST-A (s)	20.1 (0.69)	22.5 (0.85)	20.6 (071)	20.4 (0.88)	3.04	0.05	0.02	0.47	0.69
RT CST-B (s)	24.1 (0.77)	23.7 (0.95)	23.4 (0.81)	23.6 (0.80)	0.22	0.88	0.70	0.40	0.64
RT CST-C (s)	28.1 (1.08)	27.8 (1.19)	26.7 (1.11)	27.8 (1.31)	0.39	0.76	0.79	0.32	0.86
Interference (CST_i_)	28.1 (4.8)	21.3 (4.9)	22.5 (4.8)	27.2 (5.1)	0.43	0.74	0.36	0.47	0.89
Digit Span Test									
Forward—correct	8.1 (0.5)	8.6 (0.4)	8.5 (0.4)	7.9 (0.4)	2.49	0.09	0.09	0.33	0.58
Backward—correct	8.8 (0.4)	8.5 (0.5)	8.5 (0.5)	8.0 (0.4)	1.76	0.19	0.32	0.41	<0.05
Balance Test									
A95—eyes open (cm)	0.09 (0.1)	0.28(0.1)	0.54 (0.1)	1.04 (0.1)	14.12	<0.001	0.07	0.001	<0.001
A95—eyes closed (cm)	0.50 (0.1)	0.64 (0.1)	0.87 (0.1)	1.18 (0.2)	6.53	<0.01	0.37	0.001	<0.01
Subjective evaluations									
Drunkenness									
Before tests	8.3 (2.1)	28.8 (4.2)	39.3 (4.5)	51.3 (4.0)	28.49	<0.001	<0.001	<0.001	<0.001
After tests	10.0 (2.0)	27.5 (4.0)	34.8 (3.7)	45.6 (4.2)	20.77	<0.001	0.001	<0.001	<0.001
Alertness	69.3 (3.2)	64.3 (3.6)	65.6 (3.6)	56.1 (4.0)	11.86	<0.001	0.09	0.23	0.001
Contentedness	79.2 (2.3)	79.5 (2.3)	80.5 (2.5)	81.0 (2.3)	0.83	0.49	0.83	0.29	0.15
Calmness	79.6 (2.7)	76.3 (3.2)	76.0 (3.4)	77.1 (2.6)	1.37	0.28	0.13	0.11	0.30

*RT* reaction time

A main effect of alcohol was found in tracking performance at the CTT (*F*_3,21_ = 5.40, *p <* 0.01). Contrast analysis revealed a decrease of tracking performance with a BAC of 0.2 g/L (*F*_1,23_ = 6.26, *p <* 0.05) and 0.8 g/L (*F*_1,23_ = 17.57, *p <* 0.001) compared with placebo. A main effect of alcohol was found for reaction time at the DAT (*F*_3,19_ = 5.71, *p <* 0.01). Contrast analysis indicated that this effect on reaction time was due to all three active alcohol conditions: with a BAC of 0.2 g/L (*F*_1,21_ = 4.40, *p <* 0.05), 0.5 g/L (*F*_1,21_ = 9.53, *p <* 0.01), and 0.8 g/L (*F*_1,21_ = 16.47, *p =* 0.001) compared with placebo. For tracking error, a trend was found (*F*_3,21_ = 2.70, *p =* 0.07).

The inverse reaction time (1/RT) at the PVT significantly differed between alcohol conditions (*F*_3,21_ = 5.74, *p <* 0.01). Contrast analysis indicated that this effect was due to both the BACs of 0.5 g/L (*F*_1,23_ = 8.46, *p* < 0.01) and 0.8 g/L (*F*_1,23_ = 14.12, *p* = 0.001) compared with placebo. Reaction time at the ANT differed between alcohol conditions (*F*_3,21_ = 9.93, *p* < 0.001). Reaction time increased with a BAC of 0.8 g/L (*F*_1,23_ = 19.11, *p* < 0.01) as compared with placebo. No main effects of alcohol were found at the three different networks.

A main effect of alcohol was found for correct responses at the DSST (*F*_3,21_ = 11.12, *p <* 0.001). Participants’ correct responses decreased significantly with a BAC of 0.8 g/L (*F*_1,23_ = 8.32, *p* < 0.01) compared with placebo.

In the PBT, values of the A95 were not normally distributed and therefore natural log-transformed (e.g., Boyle et al. 2009). Main effects of alcohol were found in both the eyes open (*F*_3,21_ = 14.12, *p* < 0.001) and eyes closed condition (*F*_3,21_ = 6.53, *p* < 0.01). In the eyes open condition, contrast analysis indicated a trend of A95 with a BAC of 0.2 g/L (*F*_3,21_ = 3.52, *p* = 0.07) and simple effects with a BAC of 0.5 g/L (*F*_1,23_ = 13.33, *p* = 0.001) and 0.8 g/L (*F*_1,23_ = 45.74, *p* < 0.001) compared with placebo. In the eyes closed condition, simple effects were found with a BAC of 0.5 g/L (*F*_1,23_ = 15.15, *p* = 0.001) and 0.8 g/L (*F*_1,23_ = 12.84, *p* < 0.01) compared with placebo.

No main effects of alcohol were found at the three subtests of the Concept Shifting Test (i.e., CST-A, CST-B, and CST-C) and the interference score and at the two subtests of Digit Span Test (i.e., forward and backward).

### Subjective evaluations

Participants’ ratings of intoxication as measured by two VAS (i.e., before and after the test battery) differed between alcohol conditions both before (*F*_3,21_ = 28.49, *p* < 0.001) and after (*F*_3,21_ = 20.77, *p <* 0.001) testing. Simple contrasts indicated that participants felt more intoxicated before testing with a BAC of 0.2 g/L (*F*_1,23_ = 17.05, *p* < 0.001), 0.5 g/L (*F*_1,23_ = 52.53, *p* < 0.001), and 0.8 g/L (*F*_1,23_ = 81.92, *p* < 0.001) compared with placebo. Simple contrasts indicated that participants felt more intoxicated after testing with a BAC of 0.2 g/L (*F*_1,23_ = 13.57, *p* ≤ 0.001), 0.5 g/L (*F*_1,23_ = 53.56, *p* ≤ 0.001), and 0.8 g/L (*F*_1,23_ = 59.03, *p* ≤ 0.001) compared with placebo (see Fig. 4d for an average of both scales). Participants’ ratings of alertness differed significantly between BAC conditions (*F*_3,21_ = 11.86, *p* < 0.001). Participants indicated decreased alertness in the 0.8 g/L condition (*F*_1,23_ = 13.85, *p* = 0.001) as compared with placebo. No main effects of alcohol were found for subjective contentedness and calmness. The answer to the question which BAC was aimed for was indicated correctly on 56 % of the occasions.

### Comparison of performance measures

A summary of the mean difference scores with 95 % confidence intervals, mean placebo-normalized *z*-scores, and Dunlap’s effect sizes (ES) is shown in Table 2.

**Table 2:** Mean difference scores with 95 % confidence intervals, mean placebo-normalized *z*-scores, and effect sizes (Dunlap’s) of the performance tests

Test	0.2 g/L		0.5 g/L		0.8 g/L		0.2 g/L	0.5 g/L	0.8 g/L	PLA versus 0.2	PLA versus 0.5	PLA versus 0.8
	Δ	95 % CI	Δ	95 % CI	Δ	95 % CI	*z*-scores					
Critical Tracking Test												
Lambda (rad/s)	−0.18	−3.33 to −0.03	+0.08	−0.21 to 0.56	−0.31	−0.47 to −0.16	0.30	0.13	0.48	0.29	0.13	0.50
Divided Attention Test												
Average error (mm)	+0.74	−0.36 to 1.85	−0.17	−1.22 to 0.87	+1.78	0.37 to 3.18	0.23	−0.05	0.47	0.21	−0.05	0.48
RT (ms)	+123	1 to 245	+116	38 to 194	+209	102 to 316	0.33	0.40	0.70	0.34	0.39	0.65
Psychomotor Vigilance Test												
1/RT (ms)	−0.09	−0.21 to 0.03	−0.14	−0.24 to −0.04	−0.31	−0.48 to −0.14	0.20	0.28	0.70	0.20	0.29	0.70
RT (ms)	+6.8	−4.6 to 18.2	+14.2	3.8 to 24.5	+24.7	12.7 to 36.8	0.15	0.31	0.64	0.16	0.31	0.68
Digit Symbol Substitution Test												
Correct responses	+1.6	−4.1 to 7.3	−1.4	−5.6 to 2.7	−6.0	−10.2 to −1.7	−0.12	0.10	0.54	−0.11	0.09	0.41
Attention Network Test												
Overall RT (ms)	+1.6	−8.2 to 11.3	+4.7	−8.0 to 17.3	+29.8	15.7 to 43.9	0.03	0.07	0.50	0.03	0.07	0.49
Alerting effect (ms)	−10.3	−26.9 to 6.4	+3.2	−15.1 to 21.4	+11.9	−10.0 to 33.8	−0.37	0.08	0.30	−0.29	0.08	0.30
Orienting effect (ms)	+0.3	−9.5 to 10.1	−5.0	−15.8 to 5.8	−0.19	−13.4 to 13.0	0.02	−0.23	−0.01	0.01	−0.22	−0.01
Conflict effect (ms)	+2.5	−8.4 to 13.3	+8.5	−12.9 to 30.0	+21.1	−2.2 to 44.4	0.06	0.20	0.51	0.06	0.21	0.51
Concept Shifting Test												
RT CST-A (s)	+2.43	0.53 to 4.33	+0.51	−0.94 to 1.97	+0.35	−1.44 to 2.14	0.60	0.15	0.08	0.55	0.14	0.06
RT CST-B (s)	−0.34	−2.13 to 1.45	−0.69	−2.37 to 0.98	−0.45	−2.42 to 1.53	−0.07	−0.18	−0.12	−0.09	−0.14	−0.03
RT CST-C (s)	−0.32	−2.77 to 2.13	−1.35	−4.12 to 1.41	−0.24	−2.99 to 2.51	−0.06	−0.25	−0.04	−0.01	−0.22	−0.04
Interference (CST_i_)	−6.8	−22.0 to 8.3	−5.67	−21.6 to 10.3	−0.96	−15.2 to 13.3	−0.29	−0.25	−0.04	−0.21	−0.23	−0.04
Digit Span Test												
Forward—correct	+0.54	−0.08 to 1.16	+0.42	−0.45 to 1.29	−0.21	−0.97 to 0.55	−0.28	−0.20	0.10	−0.22	−0.17	0.09
Backward—correct	−0.38	−1.13 to 0.38	−0.33	−1.15 to 0.48	−0.88	−1.66 to −0.09	0.17	0.13	0.41	0.18	0.14	0.42
Balance Test												
Eyes open—A95 (cm)	+0.19	−0.02 to 0.40	+0.45	0.20 to 0.71	+0.95	0.66 to 1.24	0.45	0.72	1.34	0.39	0.77	1.50
Eyes closed—A95 (cm)	+0.14	−0.17 to 0.45	+0.37	0.18 to 0.57	+0.68	0.29 to 1.07	0.23	0.69	0.89	0.22	0.62	0.96

Mean differences indicate the relative performance changes; positive *z*-scores and effect sizes indicate impairment; negative *z*-scores and effect sizes indicate an advantage in the direction of the alcohol condition

*RT* reaction time

**Fig. 3:**
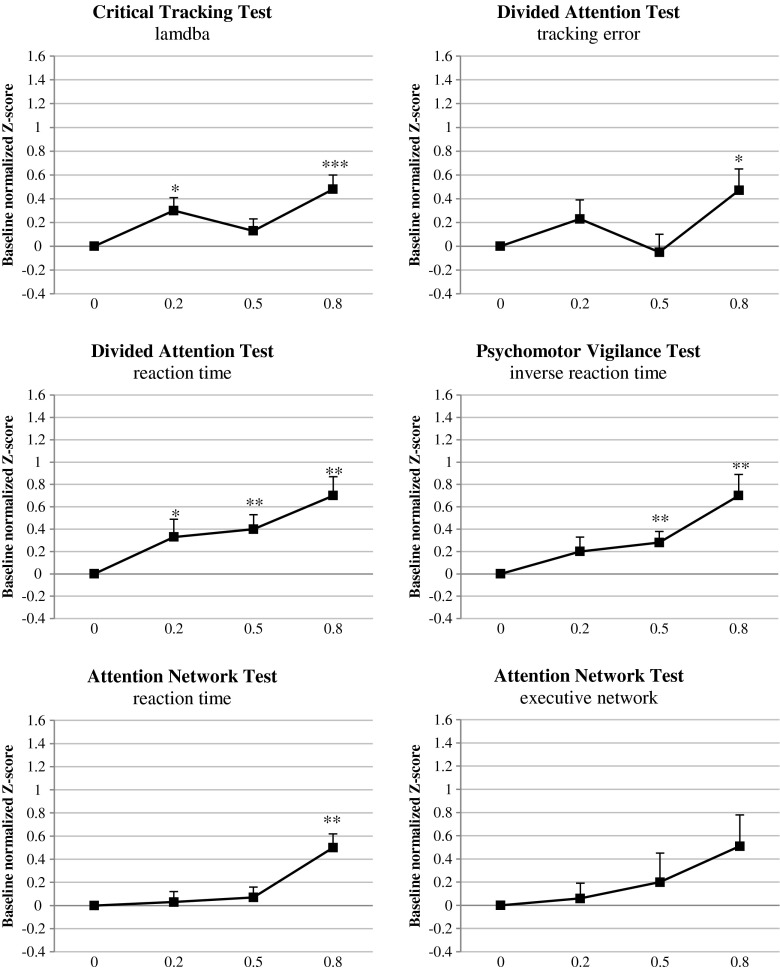
Mean baseline normalized performance of BACs 0.2, 0.5, and 0.8 g/L compared with placebo across dependent variables of the Critical Tracking Test, Divided Attention Test, Psychomotor Vigilance Test, and Attention Network Test. *p < 0.05, **p < 0.01, ***p < 0.001. *Error bars* indicate the standard error of the mean

Effect sizes and *z*-scores show that tasks and parameters differ in sensitivity to the effects of alcohol. The largest effects were found on postural balance (ES = 1.50 and 0.96, for eyes open and eyes closed, respectively), inverse reaction time in the PVT (ES = 0.70), and reaction time in the DAT (ES = 0.65) at a BAC of 0.8 g/L. Of these tests, only the reaction time in the DAT showed a significant effect of the lowest BAC (i.e., 0.2 g/L) with an ES of 0.34. All three tests show strong dose effects of alcohol, which are moderate to large for a BAC of 0.5 g/L and small to moderate for a BAC of 0.2 g/L (see Fig. 3).

**Fig. 4:**
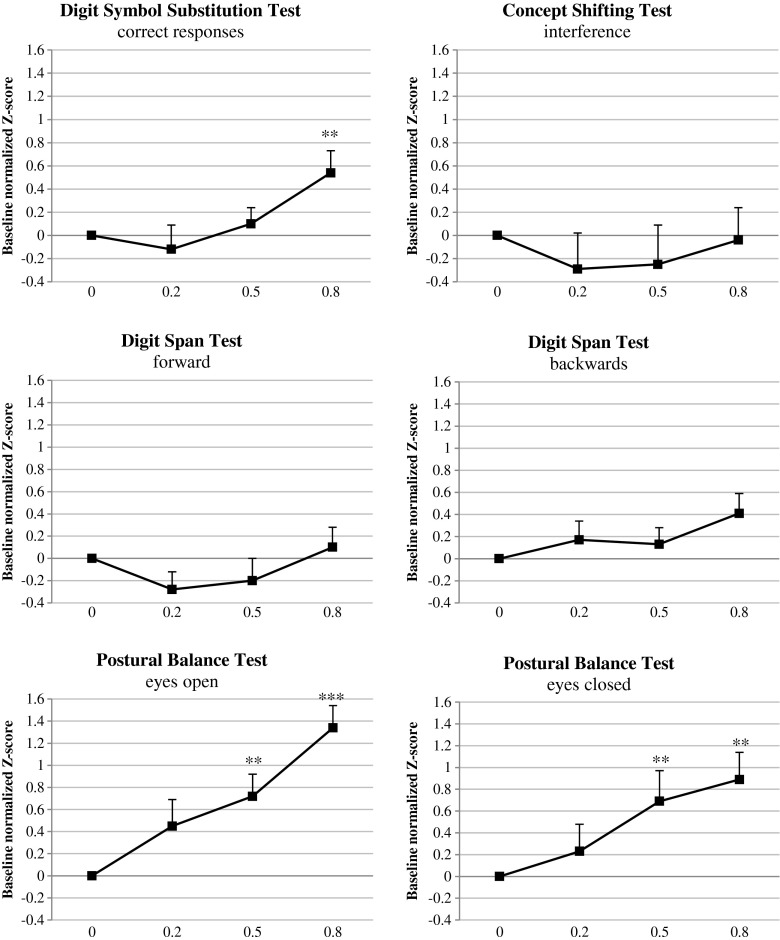
Mean baseline normalized performance of BACs 0.2, 0.5, and 0.8 g/L compared with placebo across dependent variables of the Digit Symbol Substitution Test, Concept Shifting Test, Digit Span Test, and Postural Balance Test. *p < 0.05, **p < 0.01, ***p < 0.001. *Error bars* indicate the standard error of the mean

Performance in other tests showed less or no consistent dose-dependent effects of alcohol. Performance in the DSST and ANT is only impaired at a BAC of 0.8 g/L, but not by alcohol at a BAC of 0.5 g/L or less. Performance in the CTT is impaired but does not show a consistent dose-dependent increase in the effect (see Fig. 3). Concept Shifting Test and Digit Span Test performance showed hardly any impairment (see Fig. 4).

## Discussion

The aim of this study was to determine which performance tests could be useful to measure drug-induced impairment as an initial screening tool. This was examined by assessing their ability to detect the effects of various doses of alcohol resulting in BACs between 0.0 and 0.8 g/L. Eight tests were included measuring various skills related to driving, such as psychomotor speed (CTT, ANT, PVT, DSST, DAT), divided attention (DAT), sustained attention (PVT), spatial attention (ANT), executive attention (ANT, CST), memory span (DST), and postural balance (PBT). The results showed that tasks and parameters varied in their sensitivity to the effects of alcohol.

All tests except the DST and CST showed statistically significant effects of alcohol intoxication. In terms of effects sizes, the largest and strongest dose-dependent effects of alcohol were found on performance in the PBT, PVT, and DAT. Only these tests showed significant impairment at a BAC of 0.5 g/L, the clinically relevant cutoff point as it is the legal limit for driving under the influence in most countries. Effect sizes for reaction time in the DAT and PVT were moderate (i.e., 0.39 and 0.29, respectively) at a BAC of 0.5 g/L and large (i.e., 0.65 and 0.70) at a BAC of 0.8 g/L. At the PBT with eyes open, a large effect size (i.e., 0.77) was found between placebo and a BAC of 0.5 g/L and a very large effect size (i.e., 1.50) between placebo and a BAC of 0.8 g/L.

Most of the findings of the present study regarding the effects of alcohol on performance tests are in line with previous studies. Several studies support our finding that performance in the DAT, PVT and PBT is sensitive to low or moderate BACs (e.g., Evans and Levin, 2003, 2004; Howard et al. 2007; Leung et al. 2012; McCaul et al. 2000; Moskowitz and Robinson 1988; Moskowitz and Florentino 2000; Ogden and Moskowitz 2004; Roehrs et al. 2003). Furthermore, the failure of the CST to show any effects of alcohol is in line with recent findings that performance in a similar test (i.e., TMT - part B) was only impaired at a very high BAC (i.e., 1.2 g/L), but not at lower BACs (i.e., 0.5 and 0.8 g/L) as used in the present study (Dry et al. 2012). Finally, our finding that the DSST shows impairment at a BAC of 0.8 g/L, but not below is in line with most previous studies. All studies have shown impairment at BACs of 0.8 g/L or more, but results are not consistent at BACs between 0.4 and 0.6 g/L ( Brasser et al. 2004; Brumback et al. 2007; Dumont et al., 2008; Evans and Levin 2003, 2004; Holdstock and de Wit 2001; King and Byars 2004; McCaul et al. 2000).

Regarding the CTT in the present study, impairment was found at a BAC of 0.2 and 0.8 g/L, but not at a BAC of 0.5 g/L. The failure of the CTT to show impairment at a BAC of 0.5 g/L was unexpected. Several studies found impaired tracking performance at BACs ranging from 0.4 to 0.6 g/L (Kuypers et al. 2006; Ramaekers et al. 2011b; Vermeeren and O’Hanlon 1998; Vermeeren et al. 2002). Only one study did not find impairment at a BAC of 0.64 g/L (Simons et al. 2012). Even though participants were extensively trained in the current study, a learning effect could have occurred in participants who completed the 0.5 g/L condition at the end of the second testing day. Based on previous findings, the CTT should indicate impairment at a BAC of 0.5 g/L, and therefore, this test should not be excluded as a test for initial screening. However, quantifying drug effects comparable to various BACs according to the present study should be done with caution, as no alcohol dose-dependent curve at the CTT was found in the present study.

The results of this study help further research to quantify drug effects. For example, the hypnotics gaboxadol (15 mg) and zolpidem (10 mg) taken in the middle of the night were found to increase reaction time in the DAT the next morning on average by 184 ms (Leufkens et al. 2009). These effects are comparable to the effects of a BAC of 0.8 g/L on the same test in the present study. In contrast, zopiclone (7.5 mg) taken at bedtime in the same study, increased next day reaction time in the DAT on average by 123 ms, which is comparable to a BAC of 0.5 g/L or lower according to the present study. Another study found a reaction time increase of 95 ms at the PVT for partial sleep deprivation compared with placebo, which is comparable to a BAC higher than 0.8 g/L (Bosker et al. 2010).

One of the reasons why the PVT and DAT are more sensitive to impairment may be related to their longer duration. The duration of the PVT and DAT is 10 and 12 min, respectively, whereas many other tests take no more than 2 or 3 min to complete (e.g., DST, CTT, CST, and DSST). Tests of longer duration may induce a vigilance decrement, which may enhance the impairing effects of sedative drugs. In shorter tests, a temporary increase of effort may compensate the impairing effects. Sensitivity of a test is however not only determined by its duration, as shown by the relatively small effects on performance in the longest test in the present study, the Attention Network Test, which has a duration of approximately 25 min.

One limitation of the present study is that not all available tests measuring driving-related skills could be included to compare all these tests in one study. More studies are needed comparing other tests. Recently, one other study compared six tests using dose-related effects of alcohol (Dry et al. 2012). Based on strenghts of dose-dependent effects and effect sizes, the authors concluded that the Inspection Time test (measuring information processing speed), the Self-Ordered Pointing Task (measuring working memory) and the Sustained Attention to Response Task (measuring response inhibition and cognitive flexibility) were better suited to detect impairing effects of alcohol than the TMT, the Useful Field of View test, and a problem solving test.

Furthermore, the question remains how valid laboratory tests are to assess the domains of driving as proposed by several researchers. According to Walsh et al. (2008), three core levels of behavior should be measured to predict crash risks: (1) automative behavior, (2) control behavior, and (3) executive planning behavior. Furthermore, five essential driving ability domains were indicated: (1) alertness/arousal, (2) attention and processing speed, (3) reaction time/psychomotor functions, (4) sensory-perceptual functioning, and (5) executive functions (Kay and Logan 2011). It could be argued that the DAT is a relatively complex task incorporating all aspects, whereas the PVT is a relatively simple task which may be considered to be less sensitive to deficits in executive functioning. When using the PVT, an additional test for executive function deficits may be needed to cover the most relevant domains for driving. The validity of the PBT is less clear; it is not known whether the PBT is a valid measure of actual driving and whether it can predict actual driving performance. To predict actual driving impairment, the CTT and DAT have been found to moderately predict performance at the on-the-road driving test (Ramaekers 2003; Verster and Roth 2012).

Another aspect is that effects of sedative drugs or sedative conditions (e.g., sleep deprivation) can be quantified comparable to a particular BAC. Although there are many different drugs with differing mechanisms of action, one of the most common drug effects relevant for potential driving impairment is sedation or drowsiness, which is usually associated with slowing of responses and attentional deficits. It should however be noted that sedative drugs, sleep deprivation, and alcohol can have qualitatively different effects, as has been found previously (e.g., Kleykamp et al. 2010; Tiplady et al. 2003). We are currently exploring the differential effects of sleep deprivation and sedative drugs on tests measuring driving-related skills.

In conclusion, the preferable tests for initial screening are the DAT and the PVT, as these tests were most sensitive to the impairing effects of alcohol and being considerably valid in assessing potential driving impairment because of sedation or drowsiness.
